# Parotid Tumor

**Published:** 1887-06

**Authors:** 


### Parotid Tumor.—Operation.

Victor N. Bethany, Missouri, aged seven. Admitted to hospital November 9th, 1886.

*Family History.*—On father's side history is good, but on the mother's there is a strumous tendency.

*Personal History.*—About one year ago he noticed a lump on the right side of his neck, below the level of the angle of the jaw. The tumor has continuously and rapidly increased in size. There has never been any pain associated with or in the growth.

*Present Condition.*—General health good. A large smooth tumor present on the right of the neck. It is limited anteriorly at a point about midway between the angle and symphysis of the lower jaw, extending backwards to the mastoid process. It fades off above the temporal region and reaches down to the level of the clavicle. The tumor is solid and firmly bound down by the deep cervical fascia, but appears to be free from the deep structures of the neck. The trachea is drawn toward the right side.

November 11: Clinic. Examined before the class by Professor Maclean, who believed it to be an adenoid growth. The tumor is hard, smooth, and regular in outline, and its edges well defined. As it does not seem to involve the important deep structure of the neck it must be regarded as a case favorable for operation. The patient being placed under the influence of chloroform, an incision over three inches in length was made over the tumor and through the sterno-cleido-mastoid muscle which was spread out over the growth. The surrounding tissues were freed from the tumor by stripping them up with the finger, and was rapidly accomplished until the under portion of the tumor was reached, where the tissue of the growth blended with the sheath of the large blood-vessels. At this point it required a very careful dissection to free the tumor without injuring the internal jugular vein and common carotid artery. When this large mass was taken away a smaller tumor, the size of a hen's egg, and a number of enlarged glands were exposed ; these were in turn removed. The great vessels with the pneumogastric nerve were exposed to the extent of several inches, the common sheath having been dissected away. The amount of hæmorrhage was exceedingly small, there being no blood-vessels of any size divided, but a branch of the spinal accessory nerve and several small branches of the cervical plexus were torn. The wound was washed with bichloride solution (one to two thousand), a rubber drainage tube placed in the cavity, and the wound closed with carbolized silk sutures, excepting at the most dependent part where the tube projected, and dressed antiseptically.

November 16 : The sutures were removed this morning, and the patient shown at clinic in the afternoon.

November 22 : The wound practically healed and patient permitted to return home

Note.—The temperature never rose above the normal; highest pulse, 92.

